# Supplementary material for: Reactivating hippocampal-mediated memories during reconsolidation to disrupt fear
Source: Nat Commun. 2022 Sep 12;13:4733. doi: 10.1038/s41467-022-32246-8 (PMC9468169; doi:10.1038/s41467-022-32246-8)

SUPPLEMENTARY INFORMATION

TITLE: Reactivating Hippocampal-Mediated Memories During Reconsolidation to Disrupt Fear

AUTHOR LIST:

Stephanie L. Grella[1,2], Amanda H. Fortin[1], Evan Ruesch[1], John H. Bladon[1,3], Leanna F. Reynolds[1], Abby Gross[1], Monika Shpokayte[1], Christine Cincotta[1], Yosif Zaki[4], & Steve Ramirez[1]

AFFILIATIONS

[1]Department of Psychological & Brain Sciences, Boston University, Boston MA, 02215; [2]Department of Psychology, Loyola University Chicago, Chicago, IL, 60660, [3]Department of Psychology, Brandeis University, Waltham, MA, 02453; [4]Department of Neuroscience, Icahn School of Medicine at Mount Sinai, New York, NY, 10029.

CORRESPONDING AUTHOR: Steve Ramirez: dvsteve@bu.edu

Words in abstract: (156 words)

Words in main text (excl abstract, methods, references & figure legends) (6947 words)

Number of main figures: (8); **Number of supplemental figures:** (5)

Number of references: excluding methods only refs (5): (75)

KEYWORDS: reconsolidation, memory-updating, engram, dentate gyrus, fear conditioning, PTSD, optogenetics, valence, ensemble

Figure Legends

Supplementary Fig. 1. | Fear Acquisition and Post-Reactivation Long-Term Memory.

Mice subject to a 4-shock FC protocol exhibited a stepwise function of fear with relatively little freezing prior to the first shock, and gradually higher levels of freezing with the presentation of each successive shock **Left panels:** two-way RM ANOVAs; **a,** F(4,144)=138.7, P<0.0001; **b,** F(4,192)=205.4, P<0.0001; **c,** F(4,184)=198, P<0.0001; **d,** F(4,148)=122.9, P<0.0001; **e,** F(4,184)=174.9, P<0.0001; **f,** F(4,92)=98.99, P<0.0001; **g,** F(4,64)=23.48, P<0.0001; **h,** F(4,40)=47.34, P<0.0001. We also examined the rate of EXT learning across the first 3 min of day 1 to the last 3 min of day 2. **Right panels:** three-way RM ANOVAs; **a,** All mice extinguished across time (F(1,34)=87.63, P<0.0001); however, mice in the neutral groups (clean cage) and the positive (female exposure) eYFP group showed higher freezing in the first 3 min (F(1,34)=5.328, P=0.0272, Virus; F(2,34)=4.392, P=0.0201, Valence) when stimulation occurred in the latter half of the session. **b,** ChR2 mice showed less freezing in the first 3 min with stimulation in the first half of the session (F(1,48)=54.44, P<0.0001, Time; F(1,48)=11.37, P=0.0015, Virus) in the reinstatement experiment **c,** but not the spontaneous recovery experiment (F(1,46)=6.364, P<0.0152). **d,** ChR2 mice in the positive (cocaine) and neutral (homecage) groups froze less during the first 3 min (F(1,37)=13.59, P=0.0007, Valence x Time). **e,** Mice that received VTA stimulation, despite whether they received reactivation of a VTA stimulation memory, froze less throughout EXT (F(1,46)=4.231, P=0.0454, DG Virus x Time; F(1,46)=14.05, P=0.0005, VTA Virus) **f,** For randomly labeled dDG cells, ChR2 mice in both diluted and undiluted groups froze less than eYFP mice (F(1,23)=14.96, P=0.0008, Time, F(1,23)=11.38, P=0.0026, Virus). **g,** There was a similar effect with this viral strategy for mice trained on a spatial task where ChR2 mice froze less than eYFP mice in the first 3 min

(F(1,16)=5.86, P=0.0277, Time x Virus). **h,** and throughout EXT in the experiment where we combined viral strategies **(**F(1,11)=39.04, P<0.0001). Data are represented as means ± s.e.m. *P<0.05, **P<0.01, ***P<0.005, ****P<0.00. dDG dorsal dentate gyrus, EXT: extinction, S1-S4: shock 1-4, VTA: ventral tegmental area. Source data are provided as a Source Data file.

Supplementary Fig. 2. | Considering Temporal Dynamics. To better understand why optical stimulation was more effective when given in the first half of the recall session (F10) compared to the latter half (L10), we ran an additional group that received stimulation in the middle (M10). These mice received no stimulation in the first or last 5 min. **a,** Viral strategy and experimental design. dDG cells encoding a positive experience (female exposure) were tagged off-DOX (orange). **b-g,** Mice showed greater freezing post-shock following FC (three-way RM ANOVA, F(1,31)=377.6, P<0.0001; two-way RM ANOVAs, Stim 10-20: F(4,36)=32.88, P<0.0001, Stim 5-15: F(4,28)=59.06, P<0.0001, Stim 0-10: F(4,60)=68.35, P<0.0001). **h-j,** During recall, we saw less freezing for the L10 ChR2 group (two-way RM ANOVA, F(3,27)=5.733, P<0.0036, Time x Virus). More specifically, we saw differences between ChR2 and eYFP mice in the 10-15 min interval (P=0.0005) and 15-20 min interval (P=0.0141). We also saw less freezing for the F10 ChR2 group (F(1,15)=17.84, P<0.0007). Specifically, between ChR2 and eYFP mice in the 5-10 min interval (P=0.0047), 10-15 min interval (P=0.0044) and 15-20 min interval (P=0.0254). However, there were no group differences in the M10 condition during recall. **h-j,** In the first 3 min of EXT, all ChR2 groups froze less than eYFP groups (three-way RM ANOVA, F(1,31)=5.603, P=0.0244, Time x Virus). While there was only a significant difference between F10 ChR2 and eYFP mice (P=0.0414), the ChR2 groups in both the F10, and the M10 conditions started EXT with lower freezing levels as there was not a significant difference in those groups

across time. **n-p,** We saw a general decrease in freezing in ChR2 compared to eYFP groups during EXT (three-way RM ANOVA, F(1,31)=77.11, P<0.0001, Time; F(1,31)=15.68, P=0.0004, Virus). **q-s,** We saw low levels of freezing and no group differences during IS. **t-v,** During RE, ChR2 mice in the F10 (P<0.0001), and the M10 conditions (P=0.0205) froze less than eYFP controls (two-way ANOVA, F(1,31)=37.40, P<0.0001) and from **t-v,** IS to RE (three-way ANOVA, F(1,31)=50.39, P<0.0001, Time x Virus; ChR2 vs eYFP: F10 (P<0.0001) M10 (P=0.0021). EXT: extinction, IS: immediate shock, RE: reinstatement, Source data are provided as a Source Data file.

Supplementary Fig. 3. | Decreases in Fear are Not Due to Virus or Light Alone. Optical stimulation (in the absence of the ChR2 protein), or the presence of ChR2 (in the absence of light) alone, does not account for the diminished freezing observed throughout this study. Both ChR2 and laser stimulation are required. **a,** Viral strategy and experimental design. dDG cells encoding a positive experience (female exposure) were tagged off-DOX (orange). **b-c,** Mice demonstrated greater freezing post-shock following FC (two-way RM ANOVA: F(1,27)=595.2, P<0.0001; F(4,108)=142.6, P<0.0001). **d,** During recall, only mice in the ChR2-Laser On group froze less compared to other groups throughout the session (three-way RM ANOVA: F(1,27)=9.111, P<0.0055, Time; F(1,27)=6.342, P<0.018, Light x Virus). Specifically, the ChR2-Laser On group showed less freezing compared to the ChR2-Laser Off (P=0.0356; P=0.0451) and No Virus – Laser Off groups (P=0.0024; P=0.0349) in both halves of the session and compared to the ChR2-eYFP group (P=00.0028) in the latter half. **e,** All mice extinguished fear responding (three-way RM ANOVA: F(1,27)=74.93, P<0.0001); however, mice in the ChR2-Laser On group began EXT already exhibiting lower freezing (within the first 3 min)

(three-way RM ANOVA: F(1,27)=4.614, P<0.0408); ChR2-Laser On vs. eYFP-Laser On: P=0.02), **f,** which persisted throughout the session (three-way RM ANOVA: F(1,27)=77.49, P<0.0001, Time; F(1,27)=4.221, P<0.0497, Light x ChR2 Virus; ChR2-Laser On vs. eYFP-Laser On P=0.0355). **g,** No group differences were observed during IS. **h,** During RE, ChR2-Laser On mice showed reduced fear compared to other groups (two-way ANOVA: F(1,27)=18.34, P=-0.0002, Light x Virus; ChR2-Laser On vs. eYFP-Laser On P<0.0001, ChR2-Laser On vs. ChR2-Laser Off P=0.0129, ChR2-Laser On vs. No Virus-Laser Off P=0.029). **h,** From IS to RE, all mice increased freezing (three-way RM ANOVA: F(1,27)=17.25, P=-0.0003, Light x Virus x Time) with the lowest freezing exhibited in ChR2-Laser On mice **(**ChR2-Laser On vs. eYFP-Laser On P<0.0001, ChR2-Laser On vs. ChR2-Laser Off P=0.0018, ChR2-Laser On vs. No Virus-Laser Off P=0.0072, eYFP-Laser On **vs.** No Virus-Laser Off P=0.019). Data are represented as means ± s.e.m. *P<0.05, **P<0.01, ***P<0.005, ****P<0.00. dDG: dorsal dentate gyrus, DOX: doxycycline, EXT: extinction, IS: immediate shock, RE: reinstatement. Source data are provided as a Source Data file.

Supplementary Fig. 4. | Fear Memory-Updating Via Artificial Stimulation of a Positive Memory is Context Specific. To assess whether fear memory modulation depends on fear memory activation, we included two groups that received optical reactivation of a tagged positive memory (female exposure) in a context other than the conditioning chamber (context D). When compared to the original groups given stimulation within the conditioning context (F10 or L10) we found that fear memory-updating via artificial stimulation of a positive memory is indeed dependent on activation of the fear memory, in this case through exposure to the conditioning context. **a,** Viral strategy and experimental design. dDG cells encoding a positive

experience (female exposure) were tagged off-DOX (orange). **b,** Mice showed greater freezing post-shock following FC (context A) (three-way RM ANOVA: F(1,47)=572, P<0.0001). **c,** F10 reactivation of the positive memory occurring in context A produced decreases in freezing in ChR2 mice compared to eYFP controls (three-way RM ANOVA: F(3,48)=3.419, P=0.0245, Group x Time; F(3,48)=6.637, P=0.0008, Group x Virus; 0-10: SR – P=0.0426, 0-20: SR – P=0.0089, RE – P=0.0019). However, freezing was not decreased in ChR2 groups that received positive memory stimulation in the latter half of the session (L10) or in a context D. During the last half of the recall session, ChR2 mice stimulated in a context D froze more than all other ChR2 groups (Last half: P=0.0204; First half-RE: P=0.0074; First half-SR: P=0.0078). **d,** ChR2 mice stimulated in context D continued to show similar levels of freezing compared to eYFP controls throughout extinction and **e,** during tests of the re-emergence of fear (reinstatement / spontaneous recovery), only F10 mice that received stimulation in context A expressed reduced freezing (two-way ANOVA: F(3,47)=3.349, P=0.0267, Group; F(1,47)=36.35, P<0.0001, Virus; First half-RE: P<0.0001; First half-SR: P=0.0018). All data are represented as means ± s.e.m. *P<0.05, **P<0.01, ***P<0.005, ****P<0.00. dDG: dorsal dentate gyrus, DOX: doxycycline, F10: stimulation in the first half of the session, L10: stimulation in the last half of the session. Source data are provided as a Source Data file.

Supplementary Fig. 5. | Representative images from each group. a-b, Representative images for dDG cells encoding heterogeneously-valenced behavioral epochs (positive, neutral, and negative experiences labeled with AAV9-c-Fos-tTA-TRE-eYFP. Counterstain DAPI (blue), eYFP (green arrows), c-Fos (red arrows), overlaps (yellow arrows). **c,** Representative images for VTA neurons (left hemisphere) labeled with AAV5-Ef1a-DIO-(hChR2-H134R)-eYFP (green)

and co-localized with tyrosine hydroxylase (TH, red), overlaps (yellow), counterstain DAPI (blue). **d-e,** Representative images for dDG cells randomly labeled with undiluted and diluted AAV5-CaMKIIa-(hChR2-H134R)-eYFP. Counterstain DAPI (blue), eYFP (green arrows), c-Fos (red arrows), overlaps (yellow arrows). dDG: dorsal dentate gyrus, SNc: substantia nigra pars compacta, SNr: substantia nigra pars reticulata, TH: tyrosine hydroxylase, VTA: ventral tegmental area. Source data are provided as a Source Data file.

Supplemental Fig. 1

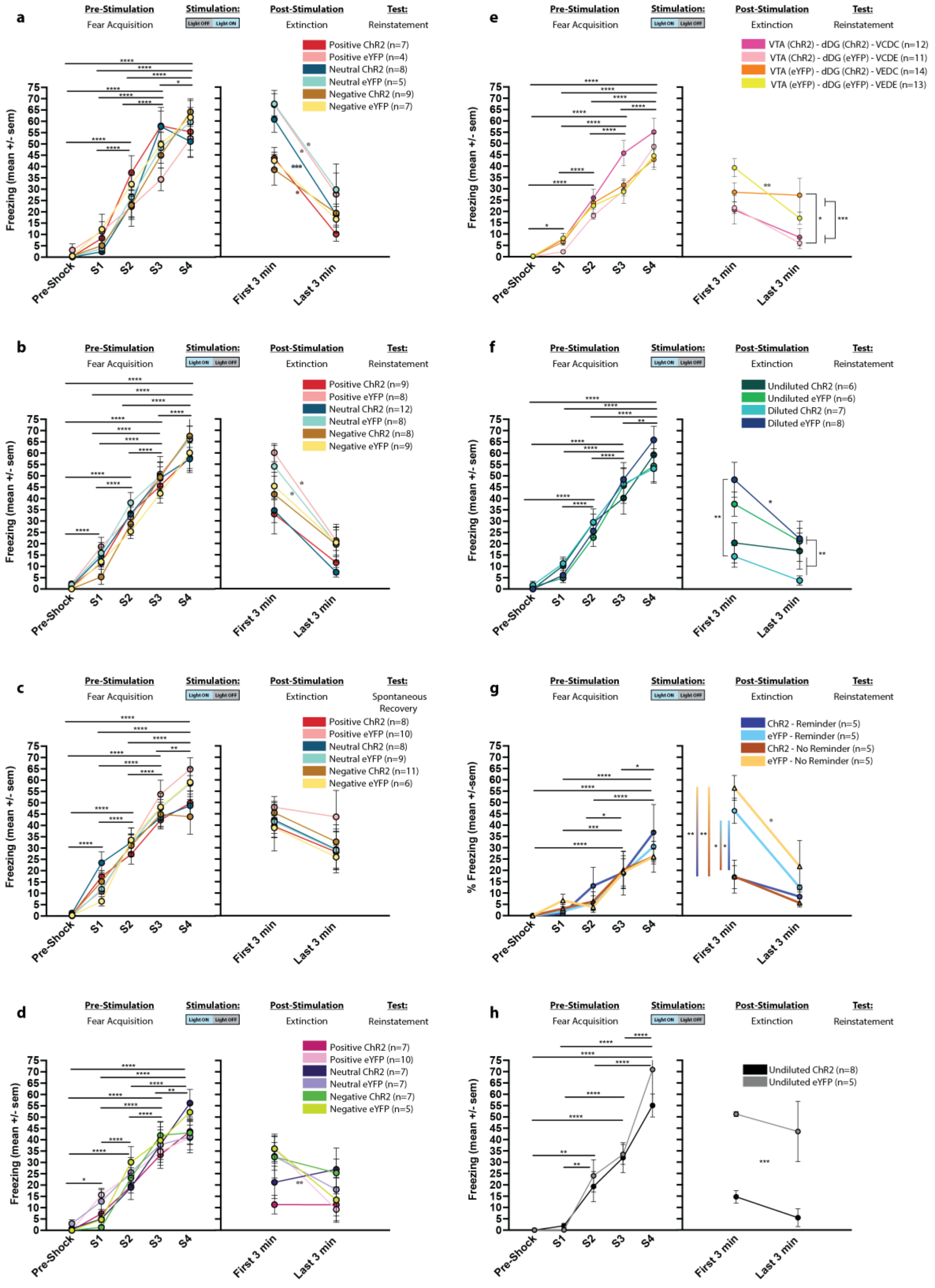

Supplemental Fig. 2

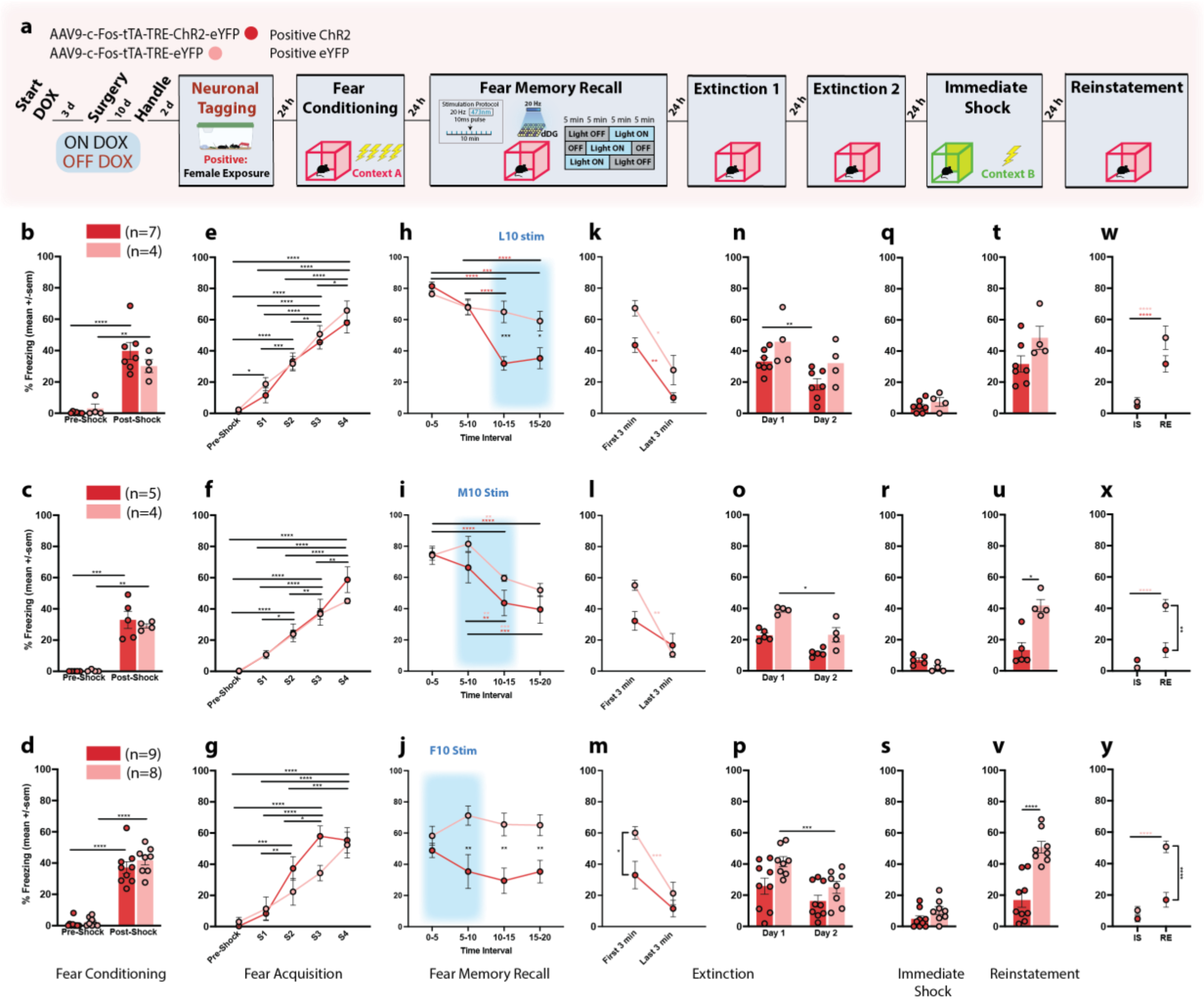

Supplemental Fig. 3

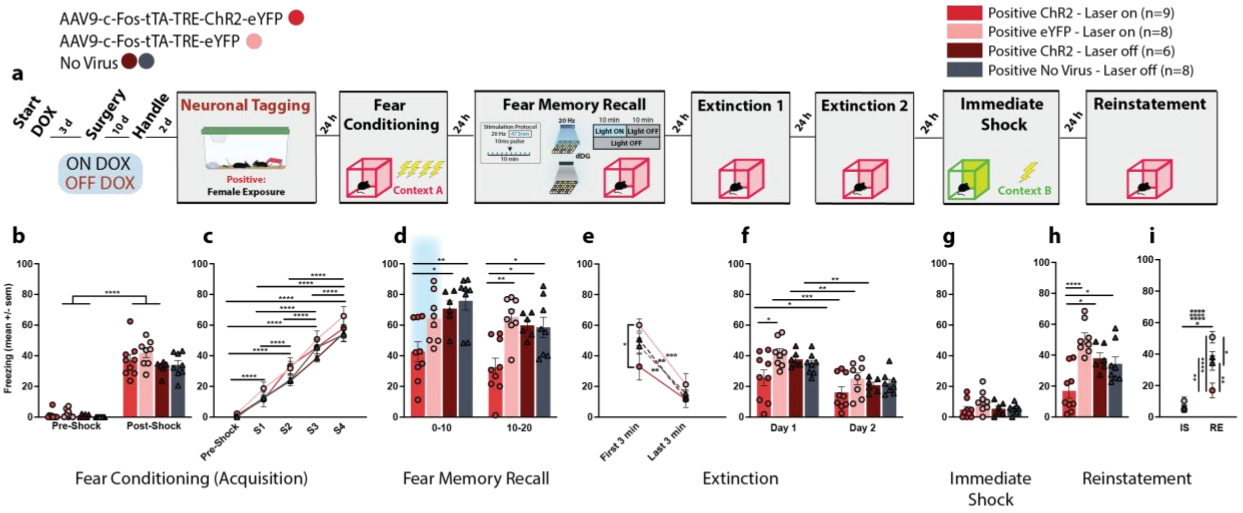

Supplemental Fig. 4

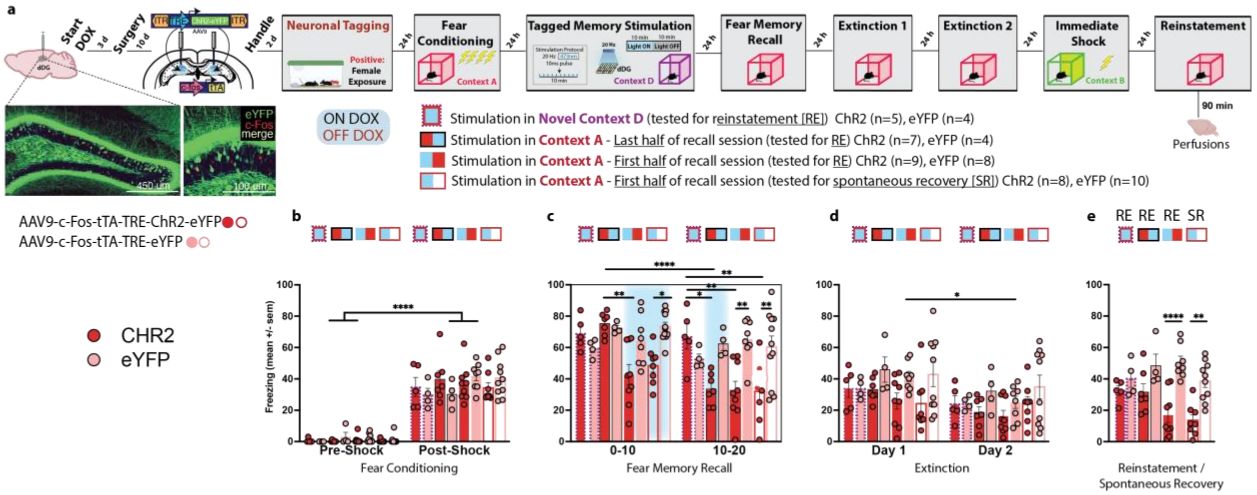

Supplemental Fig. 5

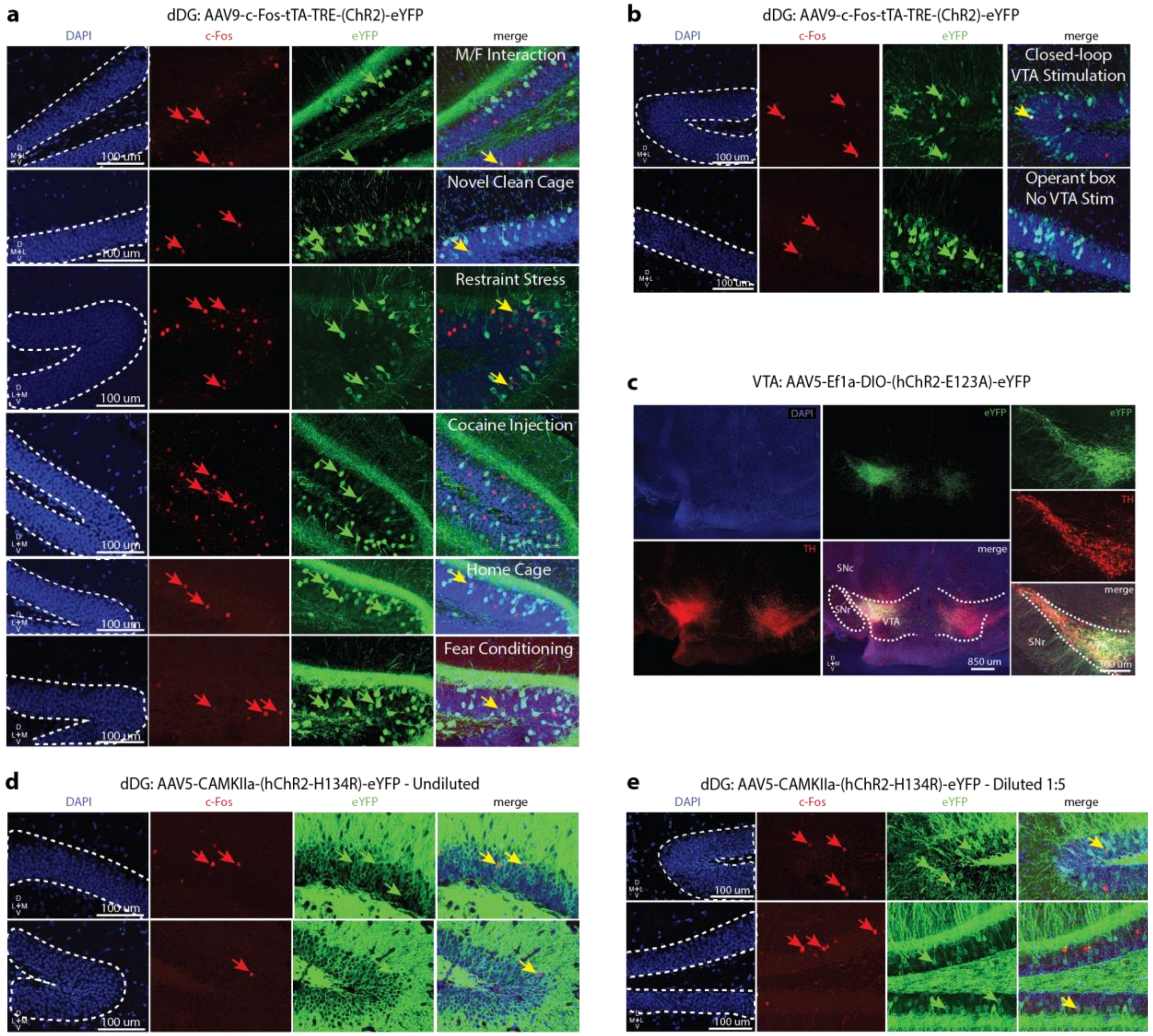

Supplement: Supplementary file 1 — Supplementary Information [file 41467_2022_32246_MOESM1_ESM.pdf]
