# Supplementary material for: Reactivating hippocampal-mediated memories during reconsolidation to disrupt fear
Source: Nat Commun. 2022 Sep 12;13:4733. doi: 10.1038/s41467-022-32246-8 (PMC9468169; doi:10.1038/s41467-022-32246-8)
Supplement: Supplementary file 3 — Reporting Summary [file 41467_2022_32246_MOESM3_ESM.pdf]

nature portfolio

Corresponding author(s): Steve Ramirez dvsteve@bu.edu

Last updated by author(s): Jul 7, 2022

Reporting Summary

Nature Portfolio wishes to improve the reproducibility of the work that we publish. This form provides structure for consistency and transparency in reporting. For further information on Nature Portfolio policies, see our Editorial Policies and the Editorial Policy Checklist.

Statistics

For all statistical analyses, confirm that the following items are present in the figure legend, table legend, main text, or Methods section.

n/a	Confirmed	
☐	☒	The exact sample size (*n*) for each experimental group/condition, given as a discrete number and unit of measurement
☐	☒	A statement on whether measurements were taken from distinct samples or whether the same sample was measured repeatedly
☐	☒	The statistical test(s) used AND whether they are one- or two-sided<br>*Only common tests should be described solely by name; describe more complex techniques in the Methods section.*
☒	☐	A description of all covariates tested
☐	☒	A description of any assumptions or corrections, such as tests of normality and adjustment for multiple comparisons
☐	☒	A full description of the statistical parameters including central tendency (e.g. means) or other basic estimates (e.g. regression coefficient) AND variation (e.g. standard deviation) or associated estimates of uncertainty (e.g. confidence intervals)
☐	☒	For null hypothesis testing, the test statistic (e.g. F, t, r) with confidence intervals, effect sizes, degrees of freedom and P value noted<br>*Give P values as exact values whenever suitable.*
☒	☐	For Bayesian analysis, information on the choice of priors and Markov chain Monte Carlo settings
☒	☐	For hierarchical and complex designs, identification of the appropriate level for tests and full reporting of outcomes
☒	☐	Estimates of effect sizes (e.g. Cohen's *d*, Pearson's *r*), indicating how they were calculated

Our web collection on statistics for biologists contains articles on many of the points above.

Software and code

Policy information about availability of computer code

Data collection	Freezing levels were collected automatically using Freeze Frame (Actimetrics version 5.105) in Colbourn operant chambers for all days except recall where they were done manually. All fluorescent images were obtained using a Zeiss confocal microscope (LSM-800) using Zen Blue 2.3 software. Cell counts were done manually using Image J / Fiji software (version 2.1.0). VTA self-stimulation was conducted using custom Matlab code (version R2021a). Optical stimulation was conducted using Doric software (Doric Neuroscience Studio version 5.3.3.14).
Data analysis	Data were analyzed using parametric statistics (Graphpad Prism version 9.2.0) following tests of normality and homogeneity of variances.

For manuscripts utilizing custom algorithms or software that are central to the research but not yet described in published literature, software must be made available to editors and reviewers. We strongly encourage code deposition in a community repository (e.g. GitHub). See the Nature Portfolio guidelines for submitting code & software for further information.

Data

Policy information about availability of data

All manuscripts must include a data availability statement. This statement should provide the following information, where applicable:

- Accession codes, unique identifiers, or web links for publicly available datasets
- A description of any restrictions on data availability
- For clinical datasets or third party data, please ensure that the statement adheres to our policy

The raw data supporting the findings from this study are available in the Source Data file. The code used for the operant experiment is provided via Github (https://github.com/bladonjay/RamirezLabCode).

Human research participants

Policy information about studies involving human research participants and Sex and Gender in Research.

Reporting on sex and gender	N/A
Population characteristics	N/A
Recruitment	N/A
Ethics oversight	N/A

Note that full information on the approval of the study protocol must also be provided in the manuscript.

Field-specific reporting

Please select the one below that is the best fit for your research. If you are not sure, read the appropriate sections before making your selection.

☒ Life sciences ☐ Behavioural & social sciences ☐ Ecological, evolutionary & environmental sciences

For a reference copy of the document with all sections, see nature.com/documents/nr-reporting-summary-flat.pdf

Life sciences study design

All studies must disclose on these points even when the disclosure is negative.

Sample size	No statistical test was used to predetermine group sizes. Groups sizes were based on what is standard for the field and what we have used in previous studies (Grella et al., 2021; Ramirez et al., 2013; Liu et al., 2014; Chen et al., 2019; Doucette et al., 2020; Ramirez et al., 2015
Data exclusions	Mice were excluded if viral injections were mistargeted or if they were euthanized for health reasons (e.g., head caps came off, mice lethargic / not grooming, lost too much weight after surgery etc.) One cage was excluded because mice were overly aggressive.
Replication	With the exception of the last test (spontaneous recovery rather than reinstatement) The groups in the experiment in figure 1n-s are a replicates of the groups in the experiment depicted in figure 1a-g and the results were similar. Similarly, the experimental groups that received the undiluted virus in Figure 4 were replicated in Figure 5 with the addition of training on the maze, and in Figure 6 with the addition of tagging the fear memory. These were not exact replicates but they used a very similar design and the results were consistent throughout. For cell counts in Figure 7, left and right hemisphere counts were combined. The data in fig 7g and h are reported twice, enlarged in fig 7 i-l. For supplemental figures 2-4, the positive ChR2 and eYFP groups that received optical stimulation in the first half of the recall session are the same groups from Figure 1h-m.
Randomization	All animals were randomly assigned to experimental groups upon arrival. During behavioural testing, order was counterbalanced.
Blinding	All tests were run blind to condition / group assignments. Cell counts and video scoring were also run blind.

Reporting for specific materials, systems and methods

We require information from authors about some types of materials, experimental systems and methods used in many studies. Here, indicate whether each material, system or method listed is relevant to your study. If you are not sure if a list item applies to your research, read the appropriate section before selecting a response.

Materials & experimental systems

n/a	Involved in the study	
☐	☒	Antibodies
☒	☐	Eukaryotic cell lines
☒	☐	Palaeontology and archaeology
☐	☒	Animals and other organisms
☒	☐	Clinical data
☒	☐	Dual use research of concern

Methods

n/a	Involved in the study	
☒	☐	ChIP-seq
☒	☐	Flow cytometry
☒	☐	MRI-based neuroimaging

Antibodies

Antibodies used

Antibodies were made in 1x PBS with 2% Triton.

Primary antibodies:
(1:1000)
rabbit anti-c-Fos (SySy 226-003, Fig. 1-5, Finkelstein et al., 2022, Chen et al., 2019, Zaki et al., 2019)
rabbit anti-c-Fos (Abcam ab190289, Fig. 6]
chicken anti-GFP (Invitrogen a10262, Fig. 1-6, Chen et al., 2019, Finkelstein et al., 2022, Zaki et al., 2019)
guinea anti-RFP (SySy 390 004 Fig. 6)
(1:500)
rabbit anti-TH (Millipore AB152, Fig. 3, Cuesta et al., 2018)

Secondary antibodies:
(1:200)
Alexa 555 goat anti-rabbit (Invitrogen A21428, Fig. 1-5, Finkelstein et al., 2022; Chen et al., 2019, Zaki et al., 2019)
Alexa 488 goat anti-chicken (Invitrogen, A11039, Fig. 1-6, Finkelstein et al., 2022; Chen et al., 2019, Zaki et al., 2019)
Alexa 555 goat anti-guinea (Invitrogen, A21435, Fig. 6)
Alexa 405 anti-rabbit (Abcam ab175653, Fig. 6)

Validation

These antibodies are widely used and have specifically been used in: Finkelstein et al., 2022, Chen et al., 2019, Zaki et al., 2019, and Cuesta et al., 2018 in c57BL/6 wildtype and DAT Cre mice.

Animals and other research organisms

Policy information about studies involving animals; ARRIVE guidelines recommended for reporting animal research, and Sex and Gender in Research

Laboratory animals

Experimental animals:
wildtype male c57BL/6 mice (~39 days of age Charles River Lab (stock no 027) 20-22g upon arrival.

Mice used for positive experience:
wildtype female c57BL/6 mice (~39 days of age Charles River Lab (stock no 027) 18-20g upon arrival.

Transgenic mice:
Two DAT-IRES Cre knock-in breeding pairs (The Jackson Laboratory B6.SJL-Slc6a3tm1.1 cre (Bkmn)/J Stock No 06660 used maintain a breeding colony. Male DAT Cre mice were used as experimental animals. Females were culled or used for breeding / practice surgeries. Experimentation began when these mice were approximately 34 days old.

Mice were kept on a regular light cycle 12:12 light-dark in a temperature and humidity controlled room with the temperature set to 18-23 degrees Celsius and the humidity set to 40-60%. Mice were fed a 40mg/kg DOX diet (Bio-serv product F4159, Lot 226766) and given ad libitum access to food and water.

Wild animals

No wild animals were used in this study.

Reporting on sex

Only males were used as experimental animals in this study. We recognize that there is a significant male bias in neuroscience research by over-representation of the sole use of males in studies. We also recognize that the prevalence of PTSD is twice that in females compared to males. We originally tested our hypotheses in males because contextual fear conditioning in rodents is sexually dimorphic where males typically show higher freezing levels than females (Maren et al.; Wiltgen et al.; Yavas et al., 2021) and we wanted to make sure that our effect was large enough to warrant further experimentation. Notably, we also wanted to use female exposure as a positive stimulus and received feedback that this might not necessarily be a positive experience for the females. While it is extremely unfortunate that we did not include females in this study, we are committed to being part of the changing tides on this issue and as a result, all of our current studies do include both males and females as we agree that it is absolutely essential for enhanced scientific discovery to do so.

Field-collected samples

No field collected samples were used in this study.

Ethics oversight

Experimental procedures were conducted in accordance with protocol 2018000579 approved by the Institutional Animal Care and Use Committee at Boston University.

Note that full information on the approval of the study protocol must also be provided in the manuscript.
